# Transfer learning-based approach to individual *Apis cerana* segmentation

**DOI:** 10.1371/journal.pone.0319968

**Published:** 2025-04-16

**Authors:** Panadda Kongsilp, Unchalisa Taetragool, Orawan Duangphakdee

**Affiliations:** 1 Department of Computer Engineering, King Mongkut’s University of Technology Thonburi, Bangkok, Thailand; 2 Native Honeybee and Pollinator Research Center, Ratchaburi Campus, King Mongkut’s University of Technology Thonburi, Rang Bua, Chom Bueng, Ratchaburi, Thailand; Van Yuzuncu Yil University Faculty of Veterinary Medicine: Yuzuncu Yil Universitesi Veteriner Fakultesi, TÜRKIYE

## Abstract

Honey bees play a crucial role in natural ecosystems, mainly through their pollination services. Within a hive, they exhibit intricate social behaviors and communicate among thousands of individuals. Accurate detection and segmentation of honey bees are crucial for automated behavior analysis, as they significantly enhance object tracking and behavior recognition by yielding high-quality results. This study is specifically centered on the detection and segmentation of individual bees, particularly *Apis cerana*, within a hive environment, employing the Mask R-CNN deep learning model. We used transfer learning weights from our previously trained *Apis mellifera* model and explored data preprocessing techniques, such as brightness and contrast enhancement, to enhance model performance. Our proposed approach offers an optimal solution with a minimal dataset size and computational time while maintaining high model performance. Mean average precision (mAP) served as the evaluation metric for both detection and segmentation tasks. Our solution for *A. cerana* segmentation achieves the highest performance with a mAP of 0.728. Moreover, the number of training and validation sets was reduced by 85% compared to our previous study on the *A. mellifera* segmentation model.

## Introduction

Honey bees are the primary pollinators and important contributors to the vitality of ecosystems [1]. Southeast Asia is home to eight native honey bee species, with four of them thriving in Thailand [2]. However, there has been a worrying decline in local bee populations across Southeast Asia in recent years, notably affecting *Apis cerana* [3,4]. In response to changing environmental factors, honey bees have shown adaptive behaviors to ensure their survival and habitat [5–7]. Known for their sophisticated communication methods, they convey hive conditions through dances and acoustical signals, demonstrating their impressive adaptability and resilience [8–11]. Addressing the alarming decline of honey bees requires the initial steps of recognizing, analyzing, and understanding their behavior in their natural habitat, particularly within the hive [2].

The communication and behavior of honey bees have attracted increased attention from researchers due to their essential role in understanding natural ecosystems. In addition to their crucial function in pollination, the waggle dance behavior has been used in various research projects [12] as bioindicators and ground surveyors for assessing, monitoring, reporting, and evaluating landscape health [13, 14]. A range of techniques has been developed for observing honey bee behavior within hives, ranging from traditional manual visual analysis by humans [12,15] to recent advances in automated bee recognition and behavior analysis using computer vision [16–18].

In the field of bee behavior recognition, previous studies have mainly concentrated on detecting the position of honey bees and analyzing their movements to distinguish different behaviors [19,20]. Notably, as early as 2008, Veeraraghavan and colleagues [21] emphasized the significance of modeling the anatomy of bees and incorporating both structure and motion into tracking systems for consistent behavioral studies. Their groundbreaking study introduced a model of a bee’s shape that consisted of three sections: the head, thorax, and abdomen. However, this required users to initially identify the location of a bee’s body parts in order to guide the system for individual bee tracking and behavior recognition. In more recent years, Bozek and colleagues [18] proposed an innovative system for individual honey bee recognition and localization using deep learning, specifically a fully convolutional network (FCN) with recurrent components, for tracking dense objects in images. This system provides outputs for bee body segmentation, bee body orientation, and individual bee trajectories. Subsequently, in 2021, Bozek expanded on these advancements to enable tracking of individual bees within an entire honey bee colony [22]. These studies demonstrated notable accuracy and precision in segmenting dense objects using deep learning approaches. However, they also identified certain limitations. Firstly, the fixed shapes and sizes used for bee body segmentation impede the recognition of complex behaviors, such as honey bee dance behavior. Secondly, the FCN model’s limitation lies in semantic segmentation, a pixel-level classification task in computer vision that struggles when multiple objects of the same class are occluded [23], making it challenging to distinguish the boundaries of each object.

The transition from semantic segmentation to instance segmentation has effectively addressed this challenge by labeling instances of objects belonging to the same class [24]. Recently, instance segmentation has gained significant attention from researchers and developers. Many instance segmentation frameworks were proposed and can be classified into four categories: classification of mask proposals [25,26], detection followed by segmentation [27–29], labeling pixels followed by clustering [30,31], and dense sliding window methods [32–34]. The survey on instance segmentation by Hafiz [35] provided valuable insights into various techniques of instance segmentation, covering aspects such as structure, methods, performance, strengths, and weaknesses. For performance evaluation in this study, the most popular image datasets for instance segmentation problems, including the COCO dataset [36], Cityscapes dataset [37], and Mapillary Vistas Dataset (MVD) [38], were utilized. When weighing the benefits and drawbacks of various methods, it becomes evident that each has unique strengths and weaknesses, making the choice highly dependent on the specific tasks, problem statement, and circumstances. The study highlights that detection followed by segmentation achieves high segmentation accuracy, is relatively simple to train, offers better generalization, and is faster for both training and prediction compared to other approaches.

Recently, our research group proposed individual bee segmentation and tracking within a beehive environment through the fusion of deep learning and Kalman filter techniques [39]. In that study, we employed Mask R-CNN with a ResNet-101 backbone network to address a small colony comprising approximately 1,000 bees (*Apis mellifera*) for instance object segmentation. The key advantage lies in overcoming the constraints of semantic segmentation by allowing flexible area delineation rather than fixed shapes and sizes for bee bodies. Our research demonstrated the robust performance of Mask R-CNN for dense object segmentation, achieving a mean average precision (mAP) value of 0.85.

Motivated by the declining population of *A. cerana* and building upon the success of our previous work utilizing a flexible segmentation method [39], this study shifted its focus to *A. cerana* by applying transfer learning from *A. mellifera* detection and segmentation using the Mask R-CNN model. Mask R-CNN was selected due to its well-established balance between model complexity, training time, and computational resource requirements, making it a practical choice for this study. Its modular design, which integrates region proposal and pixel-level segmentation in a single framework, has proven effective for a wide range of object detection and segmentation tasks, including those involving occluded or densely populated scenes.

Although newer segmentation methods, such as Transformer-based architectures or DeepLabV3 + , may offer higher accuracy in some contexts, they typically require significantly greater computational power and training time. For this study, such requirements were not feasible given the constraints of available resources. By contrast, Mask R-CNN provides an efficient trade-off, allowing for accurate segmentation while maintaining a manageable computational footprint.

Furthermore, the implementation of transfer learning played a pivotal role in this study. Transfer learning (TL), which leverages pre-trained models to reduce the dataset size and annotation effort required for training, is particularly advantageous for supervised tasks in new domains [40]. This approach was especially beneficial for tackling the labor-intensive annotation process required in this study, where the task involved detecting multiple honey bee bodies in the dark, crowded environment of a hive. By reducing the dataset size needed for training, transfer learning also reduced computational time, aligning with the study’s resource constraints.

**Fig 1:**
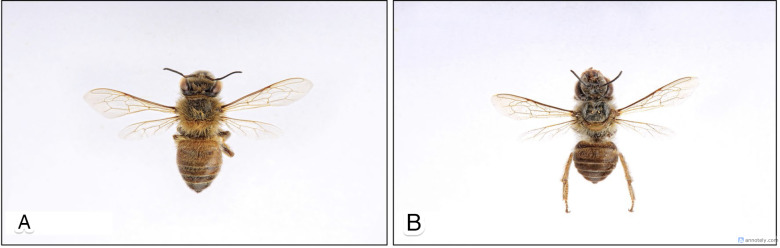
Anatomy of *Apis* species. (A) *A. mellifera*, (B) *A. cerana*.

In this study, we thus introduced a Mask R-CNN model designed to detect and segment multiple *A. cerana* specimens. We aimed to minimize the necessary number of training images by leveraging TL, drawing on insights from our previous *A. mellifera* model. Despite originating from different countries, *A. cerana* and *A. mellifera* share similar anatomical traits in terms of size and appearance. As shown in Fig 1 [41], there are noticeable differences in whole-body length and the ratio of each body section. However, they also exhibit shared biological characteristics and behaviors. The main contribution of this study is the achievement of individual *A. cerana* detection and segmentation using a minimal number of images in both the training and validation sets. As a result, the costs and time required for data annotation can be significantly reduced. Additionally, the reduced number of images can result in fewer training epochs and shorter overall training durations.

## Methods

**Fig 2:**
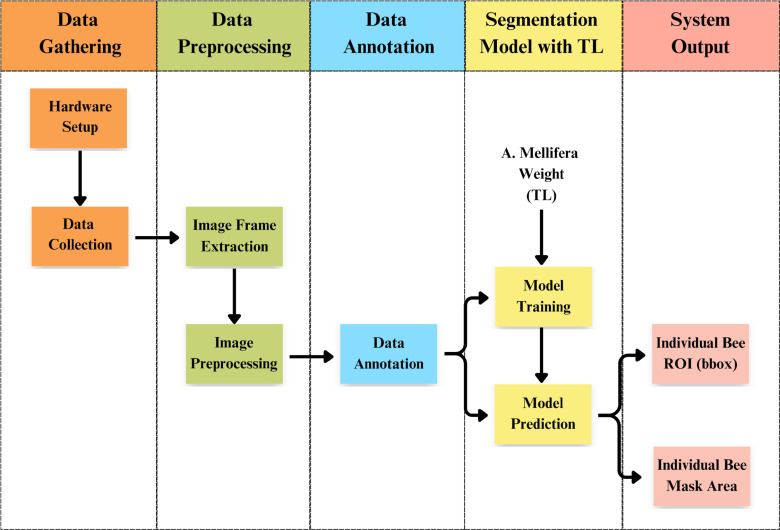
Research framework: *A. cerana* detection and segmentation.

As shown in Fig 2, our research framework consists of four main components: data gathering, data preprocessing, data annotation, and a segmentation model with TL. The first step involves data gathering, where video-format data is collected. These collected data are then processed in the second step, data preprocessing, to extract continuous image frames and improve the quality of the raw input data using various image preprocessing techniques. After preprocessing, the data annotation phase begins, where individual bee bodies are annotated to create a dataset for both model training and prediction purposes. The final and crucial component is the segmentation model. Here, we utilize TL by training our input data (*A. cerana*) with knowledge from *A. mellifera*. We employ the Mask R-CNN model with a ResNet-101 backbone. The final output of our proposed system includes the position of individual bee bodies, represented by bounding boxes, and the delineation of individual bee body regions, depicted by mask areas.

### Data Gathering

**Fig 3:**
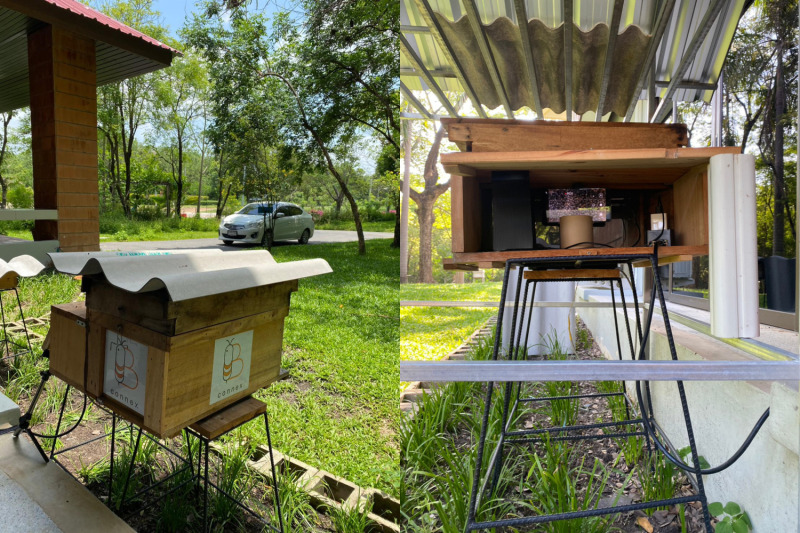
Customized observation hive.

To gather data, to gather data, we employed a customized observation hive designed to resemble a traditional *A. cerana* beehive commonly used by beekeepers in Thailand. This setup was specifically designed to minimize disruptions to the natural behavior of honey bees within their colony. The colony selected for our study consisted of three bee frames housed within the observation hive, with the observed frame strategically placed at the forefront, facing the observation area at the back of the hive. As shown in Fig 3, we added an extension box next to the traditional beehive to make beekeeping activities easier. The space between the bee colony area and the observation zone (extension box) was separated by transparent glass, allowing us to observe and collect data on bee behavior within the hive. Inside the bee colony area, we stacked three hive frames horizontally. Each frame measured 500 ×  200 mm (width ×  height). In the observation zone, we placed a smartphone with a resolution of 1920 ×  1080 pixels and a frame rate of 30 frames per second behind the transparent glass. We maintained a working distance of 5 cm (the distance from the camera to the object of interest). This setup allowed us to have a field of view measuring 450 ×  150 mm, which covered almost the entire area of one hive frame. Next to the observation area, we installed a door for easy access and setup of the image acquisition hardware. It also helped us control the ambient light (natural light) around the beehive, ensuring sufficient illumination for the video recording process.

In this study, we set up the observation hive and collected video data in the same area as our previous study [39]. The data collection began at 7 AM and ended at 5 PM, depending on the weather and sunlight availability.

### Data Preprocessing

The first step in data preparation involved extracting frames. After recording the video input data, we divided the recorded video file into a series of individual image frames. Then, we moved on to data preprocessing. In this study, we used various image processing techniques designed to improve image quality, taking into account the specific challenges posed by our problem scenarios and environmental conditions.

**Fig 4:**
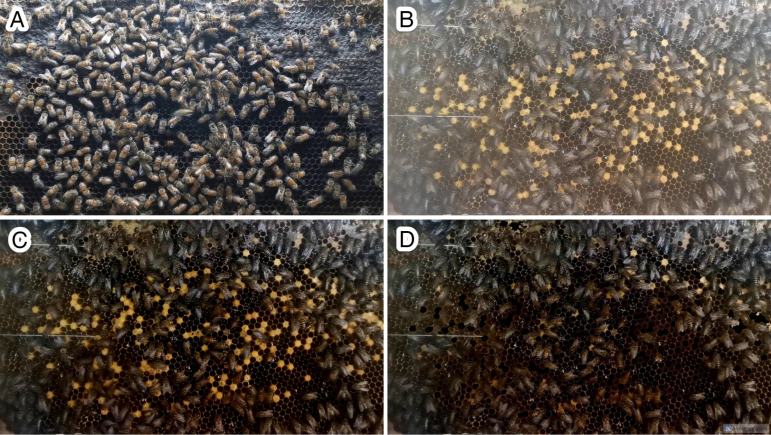
Input data. (A) Raw input data (from previous work; *A. mellifera*), (B) raw input data (*A. cerana*), (C) raw input data with brightness and contrast enhancement featuring brood, (D) raw input data with brightness and contrast enhancement in a broodless context.


When compared to the input data from our previous work with *A. mellifera* [39], as shown in Fig 4A, the image of *A. cerana* in Fig 4B appeared excessively bright and lacked contrast. To improve the quality of our input data, we initially applied image enhancement techniques, which involve manipulating images to better suit a specific application [42]. We used brightness and contrast enhancement, a process aimed at widening the dynamic range to improve visualization by maximizing information within the image [43]. This enhancement involves adjusting contrast and brightness values using alpha (*α*) and beta (*β*) parameters, which represent gain and bias, respectively. The optimal values for alpha and beta were calculated using Eq. (1), where *f*(*x*) represents the source image pixels and *g*(*x*) represents the output image pixels.

$$\begin{array}{l} g(x)=af(x)+\beta \end{array}$$ (1)

We also modified the equation for convenience, denoted as Eq. (2), where *i* and *j* represent the index of the row and column of the pixel.


..g(i,j)=α.f(i,j)+β (2)

Finally, we determined the optimized alpha and beta values for our image frame environment to be 1.3 and –110, respectively, as illustrated in Fig 4C.

Another challenge we encountered in this study was the presence of a cluttered background. There were numerous brood cells dispersed throughout the bee frame area that shared similarities in size, shape, and color with our object of interest, which is the bee body, particularly the dark yellow shade of the honey bee’s abdomen. To address this potential confusion and streamline the task for the deep learning model, we implemented a method to remove the brood cells. This involved using histogram analysis to extract the color of the brood cells and then using masking techniques to restrict their shape and area, isolating the region of interest. Finally, we applied a thresholding function to fill the selected blob area with black color. The resulting image from this step, referred to as the “broodless” image, is shown in Fig 4D and represents the final output after the preprocessing process. This dataset consists of the training set, validation set, and test set for the Mask R-CNN model.

### Data Annotation

**Fig 5:**
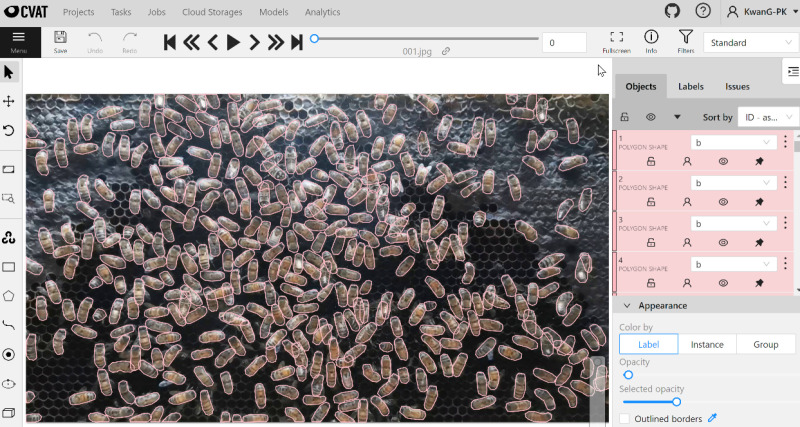
Data annotation using CVAT for Mask R-CNN training and testing.

Data annotation is a crucial process for supervised machine learning, especially when dealing with new research problems that require a fresh dataset. To prepare our dataset for model training and testing, we used the CVAT platform [44], an interactive annotation tool designed for computer vision tasks, to annotate our dataset in COCO format. Fig 5 illustrates an example of data annotation using CVAT. The annotation file, which serves as the model’s ground truth, was exported in JavaScript Object Notation format, including all the necessary attributes for multiple segmentation tasks, such as image ID, image height, image width, class ID, object instance ID, and polygon area. We defined two classes: bee body and background. In terms of annotation rules and other specifications, we followed the same procedures and conditions as in our previous work with *A. mellifera* [39].

### Segmentation Model with Transfer Learning

#### A. Model Configuration.

In this study, Mask R-CNN was implemented using the feature pyramid network and a ResNet101 backbone, following the framework and concept provided in the Matterport Mask R-CNN repository [45]. The model was built using the Keras framework with a TensorFlow backend. The development of the model was done using Python 3.7, Keras 2.14, and TensorFlow 2.14. Our system, including all model structures, training, and evaluation processes, was developed and operated within Google Colab.

#### B. Training and Testing.

The TL technique was utilized to improve model performance while addressing the challenges posed by a limited dataset size and the need to reduce training time. By transferring pretrained weights from the *A. mellifera* model, the new model leveraged object-related features, particularly bee body characteristics. This approach mitigated the necessity for extensive feature extraction from scratch.

The effectiveness of the transferred weights was dependent on the similarity between the features learned from *A. mellifera* and the requirements of the target domain, which in this study was *A. cerana*. The pretrained weights were derived by saving the weights of the *A. mellifera* model and using them as the initialization for training the *A. cerana* specific network. To adapt the model to the new dataset, specific layers (specifically mrcnn_bbox, mrcnn_mask, and anchors) were excluded from being updated during training, allowing the model to focus on learning domain-specific features while retaining generalizable knowledge from the source domain.

To improve model generalization, data augmentation techniques, specifically rotation and flipping, were applied during training. These methods artificially expanded the training dataset’s diversity, enabling the model to learn invariant features and indirectly reducing overfitting.

Hyperparameters tuning was conducted iteratively using a trial-and-error approach to optimize the model for the given dataset and settings. The final optimized hyperparameters are detailed as follows:

- Steps per epoch =  100
- Validation steps =  100
- Minimum probability value to accept a detected instance =  0.6
- Learning rate =  0.001
- Learning momentum =  0.9
- Non-max suppression threshold to filter Region Proposal Network proposals =  0.7
- Number of regions of interest kept after non-maximum suppression (training) =  6000
- Number of regions of interest kept after non-maximum suppression (prediction) =  3000
- Percent of positive regions of interest used to train classifier/mask heads =  0.33
- Number of regions of interest per image to feed to the classifier/mask heads =  1000
- Maximum number of ground truth instances to use in one image =  1000
- Maximum number of final detections =  1000

For both training and testing, the model was trained using Colab Pro + on the GPU of NVIDIA Tesla V100-PCI-E-16GB. Computation time averaged approximately 10 seconds per step, indicating the time required for each step within an epoch. For example, the training process with 100 training steps took around 1000 seconds to complete one epoch.

## Experiments

### Materials and dataset

In this study, Table 1 provides a summary of the details of our experiment. Each image frame contained approximately 350 bees. Regarding the hardware for image acquisition, the image size for both the training and testing processes was set at 1920 ×  1080 pixels. In terms of dataset preparation, we divided our data into three sets: the training set, the validation set, and the test set.

**Table 1:** Dataset for the experiment.

Data Characteristics	Mask R-CNN (Training)	Mask R-CNN (Testing)
**Image frame size**	1920 × 1080 pixels	1920 × 1080 pixels
**Number of images**	Training Set: 5 images Validate Set: 1 image	Test Set: 100 images
**Number of bees per image frame**	≈ 350 bees	≈ 350 bees

To determine the optimal training and validation image ratio for our problem (both with and without transfer learning), we evaluated model performance across three ratios: 5:1, 5:2, and 10:3. Due to resource limitations, each configuration was trained once with the same hyperparameter values as described in the previous section. A two-way ANOVA (without replication) was performed to assess the significance of both the training/validation ratio and transfer learning on the model predictive performance (measured by the mAP value). The analysis revealed a statistically significant impact of transfer learning on model performance (p-value =  0.0019). However, no statistically significant difference was observed between the training/validation ratios (p-value =  0.460), suggesting these ratios do not substantially influence model performance. Based on these findings, we decided to use a 5:1 ratio (5 images for training and 1 image for validation) for subsequent experiments. This choice minimizes resource consumption, including annotation effort, training time, GPU utilization, and storage requirements, while maintaining effective model performance.

During the testing process, we evaluated our model using 100 images from the test set. The selection of images for the training set, validation set, and test set was conducted randomly from the extracted image frames of our collected video data.

### Experimental design

To validate our proposed concept for the segmentation model of *A. cerana* using Mask R-CNN with TL from the *A. mellifera* model, we conducted a series of experiments encompassing nine scenarios to explore various conditions of interest. These experiments comprised three primary models: the *A. mellifera* model, the *A. cerana* model, and the *A. cerana* model integrated with TL from the *A. mellifera* model. Each model was further categorized into three groups based on data preprocessing techniques: raw input data, brightness and contrast enhancement, and brightness and contrast enhancement with broodless conditions. Our experimental design, as outlined in Table 2, employed datasets specified in Table 1 along with the aforementioned preprocessing techniques. Across all nine experiments, we utilized common testing sets, while training sets were exclusively employed in the *A. cerana* model and the *A. cerana* model integrated with TL (Experiments 4–9). For the *A. mellifera* model experiments (Experiments 1–3), we utilized the *A. mellifera* model from our prior study [39] and evaluated its segmentation performance using the *A. cerana* testing datasets.

**Table 2:** Experimental design for *A. cerana* segmentation evaluation.

Experiment	Model	Preprocessing Technique	Training and Validation Sets	Test Set
**1**	*Mellifera*	Raw Input Data	*Mellifera*	*Cerana*
**2**		Brightness and Contrast		
**3**		Brightness and Contrast ^+^ Broodless		
**4**	*Cerana*	Raw Input Data	*Cerana*	*Cerana*
**5**		Brightness and Contrast		
**6**		Brightness and Contrast ^+^ Broodless		
**7**	*Cerana* ^+^ TL	Raw Input Data	*Cerana*	*Cerana*
**8**		Brightness and Contrast		
**9**		Brightness and Contrast ^+^ Broodless		

### Evaluation metrics

We adopted the methodology outlined in our previous study [39] to calculate the evaluation metrics for the multiple object detection and segmentation task. Given that our proposed model focuses on segmenting multiple object instances, we used the mean Average Precision (mAP) as the primary evaluation metric, which is widely recognized for instance segmentation problems. To calculate mAP, we derived key metrics, including intersection over union (IoU), precision, and recall. IoU quantifies the overlap between the predicted and ground truth segmentation masks. Precision measure the proportion of true positive predictions among all positive predictions, while recall measures the proportion of true positive predictions among all actual positives.

**Fig 6:**
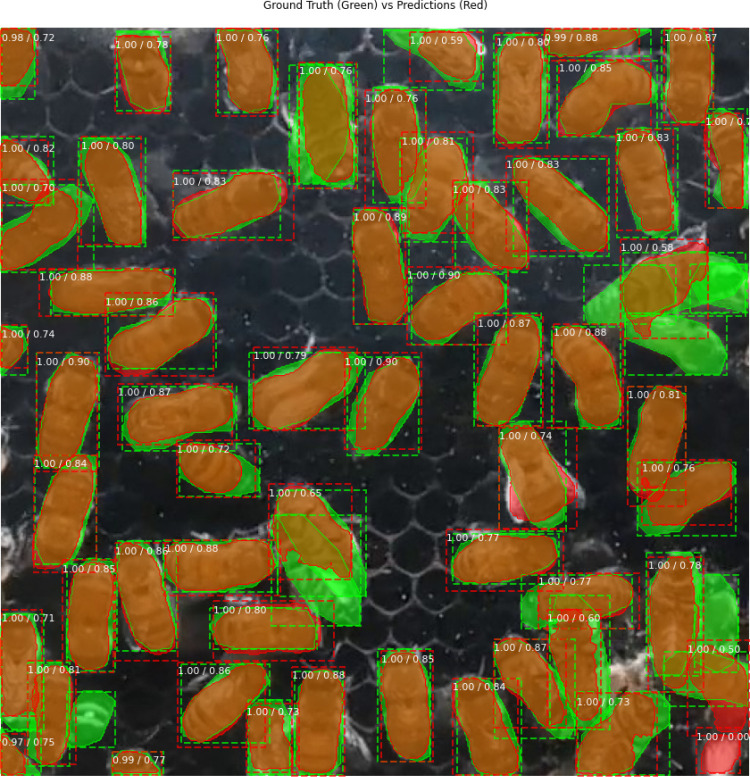
Four cases comparing the segmented objects in the prediction and the ground truth.

In this study, we categorize predictions into four cases using confusion metrics: true positive (TP), true negative (TN), false positive (FP), and false negative (FN. A prediction was considered correct if the IoU exceeded 50% (IoU threshold =  0.5). The color-coded regions in Fig 6 represent these categories: green for FN, red for FP, orange for TP (intersection green and red, when IoU ≥  0.5), and the remaining background for TN.

The mAP is calculated as the area under the precision-recall curve, reflecting the average precision (AP) across prediction. AP values range from 0 to 1, with a value of 1 indicating perfect precision and predictions. Since our model focuses on a single class—bee body—the mAP and AP values are identical in this study.

Additionally, an independent two-tailed t-test with a significance threshold of 0.05 was applied to statistically evaluate whether there was a significant difference in model predictions (segmentation output) between the model trained on the *A. mellifera* dataset and the *A. cerana* dataset without transfer learning. The t-test compared the means of the segmentation performance metrics obtained from each model to determine whether the observed differences were likely due to random variation or reflected a true effect of training data on model performance.

## Results and discussion

### Data gathering

**Fig 7:**
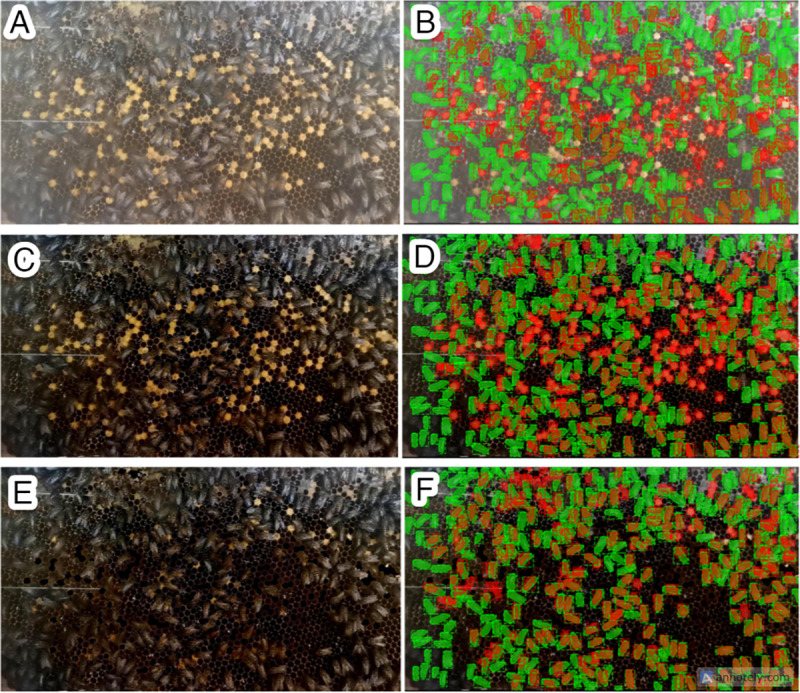
Input data and Preliminary results of multiple bee segmentation based on Mask R-CNN, illustrating predictions in orange and ground truth in green. (A) and (B) depict raw input data, (C) and (D) showcase brightness and contrast enhancement, while (E) and (F) display brightness and contrast enhancement combined with broodless preprocessing.

The raw data presented in Fig 7A highlights challenges associated with excessive brightness and low contrast, which impacted the quality of the acquired images. These limitations stemmed from the constraints of our hardware setup and our deliberate efforts to minimize disruptions to the honey bees’ natural behavior. Specifically, while natural sunlight was avoided to prevent behavioral alterations, the digital cameras used required adequate lighting for image capture. These findings underscore the need to explore alternative imaging methods, such as infrared or thermal cameras, to overcome these issues and enhance data quality in future studies.

### Data preprocessing

We implemented two preprocessing techniques to refine the quality of our input data: brightness and contrast enhancement and broodless. To fine-tune the parameters and methods for these techniques, we conducted a series of experiments and gathered preliminary results. Throughout this phase, we employed the *A. mellifera* model from our prior work [39] to assess outcomes based on mean mAP. Fig 7B illustrates the ground truth of input image (Fig 7A) with green representing each bee body and orange indicating accurate predictions. The raw input data led to numerous false negatives (green) and false positives (red), as depicted. However, upon applying brightness and contrast enhancement (Figs 7C and 7D), the number of correct predictions increased (orange), albeit accompanied by false positives within brood cells (red). To resolve this issue of misidentification between brood cells and bee bodies or red masks on brood cells, we introduced masking and thresholding (broodless). This step effectively removed brood cells from the image frame, resulting in a refined model output (Figs 7E and 7F). Initially, the mAP from raw input data was 0.035, the lowest score attributable to image quality. With brightness and contrast enhancement, the mAP improved to 0.054, indicating enhanced input data quality. The integration of brightness and contrast with broodless yielded the highest mAP value of 0.200. These preliminary findings suggest that our preprocessing techniques hold promise for enhancing segmentation task performance within our research context.

### *A. cerana* segmentation based on mask R-CNN with transfer learning

**Fig 8:**
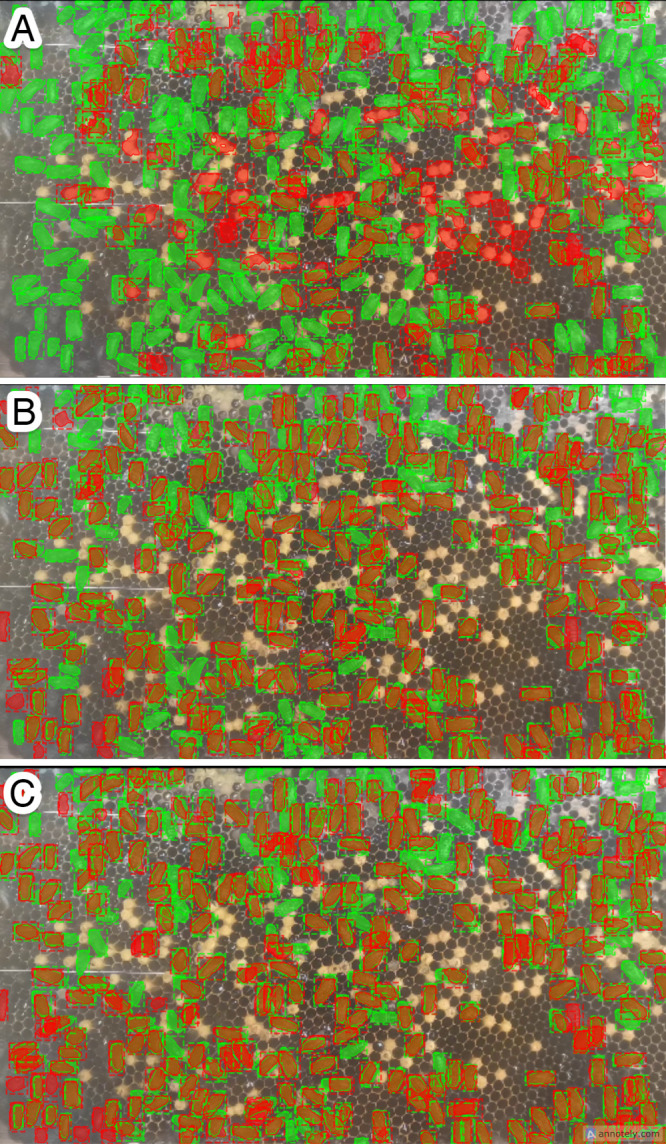
Experimental results with raw input data, illustrating predictions in orange and ground truth in green. (A) *A. mellifera* model (Experiment 1), (B) *A. cerana* model (Experiment 4), (C) *A. cerana* model with TL (Experiment 7).

**Fig 9:**
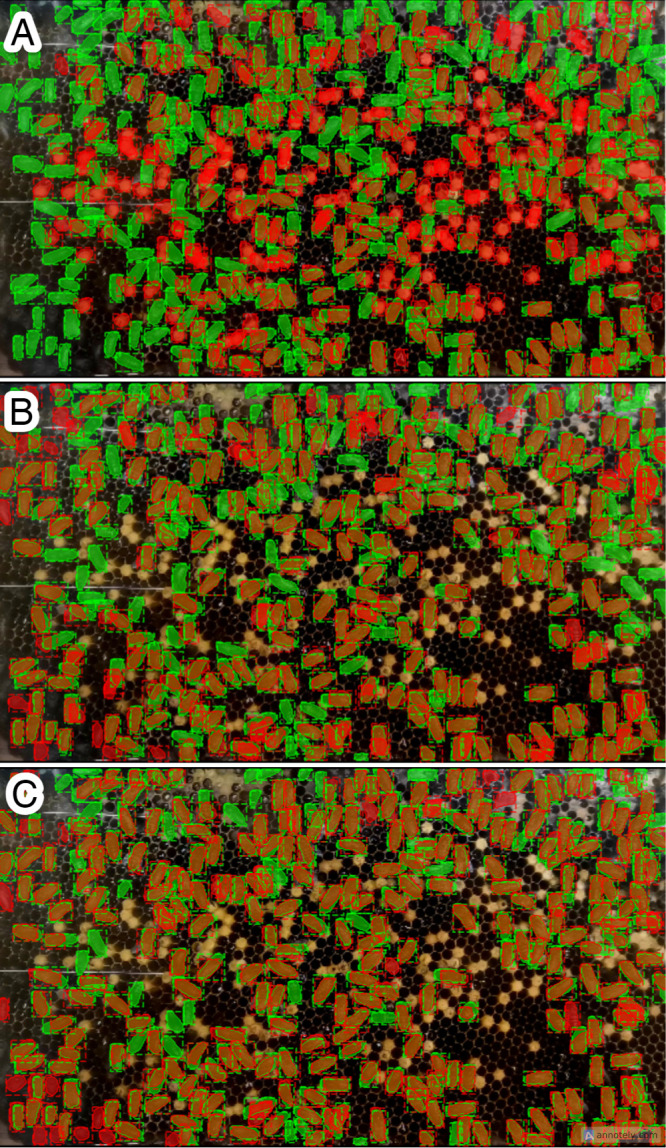
Experimental results with brightness and contrast enhancement, depicting predictions in orange and ground truth in green. (A) *A. mellifera* model (Experiment 2), (B) *A. cerana* model (Experiment 5), (C) *A. cerana* model with TL (Experiment 8).

**Fig 10:**
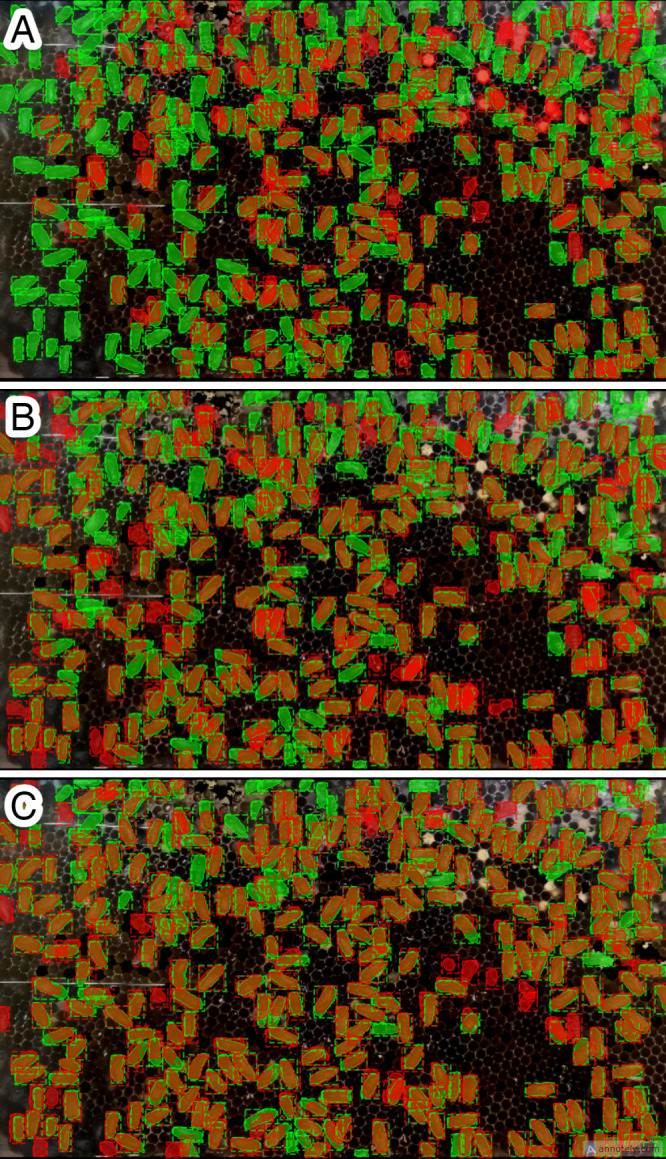
Experimental results with brightness and contrast enhancement along with broodless preprocessing, showcasing predictions in orange and ground truth in green. (A) *A. mellifera* model (Experiment 3), (B) *A. cerana* model (Experiment 6), (C) *A. cerana* model with TL (Experiment 9).

Referring to the experimental design outlined, all experiments that used the same data preprocessing technique were evaluated using identical test sets and ground truth data (annotated data). The experimental results, summarized in Table 3, show that the *A. cerana* segmentation prediction achieved the highest performance with a mAP of 0.728 using the *A. cerana* model with TL. Regarding the *A. mellifera* model (Experiments 1–3), the lowest mAP score was 0.079 with raw input data, while the highest mAP prediction was 0.248, achieved with data that employed brightness and contrast enhancement in combination with broodless conditions. As shown in Fig 4A, the *A. mellifera* observation colony used for data collection did not have any brood cells present. By eliminating brood cells, the mAP of the *A. cerana* prediction based on the *A. mellifera* model improved, reducing confusion between *A. mellifera* bodies and *A. cerana* brood cells. In contrast, the highest mAP value with the *A. cerana* model (Experiments 4–9) was obtained by using only brightness and contrast enhancement (including brood cells), resulting in 0.557 and 0.728 for the model without TL (Experiment 4) and the model with TL (Experiment 8), respectively. Since the training data and testing were both recorded from the same environment—the same bee colony and bee frame—in this case, the absence of brood cells does not affect the improvement in mAP. Figs 8–10 depict the output results of *A. cerana* segmentation prediction using different models and data preprocessing techniques.

**Table 3:** Performance evaluation of *A. cerana* segmentation based on Mask R-CNN.

Experiment	Model	Preprocessing Technique	mAP
**1**	*Mellifera*	Raw Input Data	0.079
**2**		Brightness and Contrast	0.108
**3**		Brightness and Contrast ^+^ Broodless	0.248
**4**	*Cerana*	Raw Input Data	0.557
**5**		Brightness and Contrast	0.479
**6**		Brightness and Contrast ^+^ Broodless	0.464
**7**	*Cerana* ^+^ TL	Raw Input Data	0.617
**8**		Brightness and Contrast	0.728
**9**		Brightness and Contrast ^+^ Broodless	0.716

## Conclusions

In this study, we present a method for detecting and segmenting multiple *A. cerana* bees using the Mask R-CNN with TL model. Our system aims to achieve optimal results while minimizing costs and time for both training and testing. We investigated three key aspects: the characteristics of the training set, data preprocessing techniques, and training conditions.

Regarding the training set, although *A. mellifera* and *A. cerana* have similar appearances, we conducted an independent two-tail t-test to determine whether there was a statistically significant difference in segmentation performance when the model was trained on data from *A. mellifera* (Experiments 1, 2, and 3) compared to *A. cerana* without transfer learning (Experiments 4, 5, and 6). The t-test resulted in a p-value of 0.043. Since the significance threshold was set at 0.05, the p-value being less than this threshold indicates that the difference in segmentation performance between the two groups is statistically significant. This result suggests that training the model with *A. cerana* data leads to a measurable improvement in segmentation performance compared to training with *A. mellifera* data.

Concerning data preprocessing techniques, adjusting brightness and contrast was sufficient to enhance the input data quality for *A. cerana* segmentation using the *A. cerana* model. However, when using the *A. mellifera* training model, additional techniques such as masking and thresholding (broodless) may be necessary.

The most significant findings are related to training conditions. Our experimental results show that TL increased the mAP value by 51.98%. Additionally, our proposed solution offers an alternative approach that reduces computation time and cost while maintaining high-performance prediction. Compared to our previous work on the *A. mellifera* segmentation module [39], the use of TL enabled us to reduce the training set size from thirty frames to five frames and the validation set size from ten frames to one frame. This reduction significantly decreased the time required for annotation, computational time, and the computational resources needed for model training.

However, small datasets inherently pose challenges, such as increasing the risk of overfitting and limiting generalization ability. To address these challenges, we tested different training and validation ratios and found that using five images in the training set and one image in the validation set effectively balanced resource consumption and model performance. Additionally, data augmentation techniques were applied to improve generalization and mitigate overfitting. While these measures enhanced the model’s performance, small datasets remain a limitation, particularly in their impact on generalization ability.

Future studies could investigate the effect of dataset size in more detail. Furthermore, exploring methods to address external factors, such as distractions from light sources during data acquisition, could further improve data quality. Expanding the scope of this study to include other bee species would also provide a broader understanding of the model’s adaptability across different domains.
